# Canopy Chlorophyll Density Based Index for Estimating Nitrogen Status and Predicting Grain Yield in Rice

**DOI:** 10.3389/fpls.2017.01829

**Published:** 2017-10-27

**Authors:** Xiaojun Liu, Ke Zhang, Zeyu Zhang, Qiang Cao, Zunfu Lv, Zhaofeng Yuan, Yongchao Tian, Weixing Cao, Yan Zhu

**Affiliations:** ^1^National Engineering and Technology Center for Information Agriculture, Key Laboratory for Crop System Analysis and Decision Making, Ministry of Agriculture, Jiangsu Key Laboratory for Information Agriculture, Jiangsu Collaborative Innovation Center for Modern Crop Production, Nanjing Agricultural University, Nanjing, China; ^2^Key Laboratory for Quality Improvement of Agricultural Products of Zhejiang Province, Department of Agronomy, College of Agriculture and Food Science, Zhejiang A & F University, Lin'an, China

**Keywords:** Chl, chlorophyll meter, leaf area index, nitrogen status, yield, rice

## Abstract

Canopy chlorophyll density (Chl) has a pivotal role in diagnosing crop growth and nutrition status. The purpose of this study was to develop Chl based models for estimating N status and predicting grain yield of rice (*Oryza sativa* L.) with Leaf area index (LAI) and Chlorophyll concentration of the upper leaves. Six field experiments were conducted in Jiangsu Province of East China during 2007, 2008, 2009, 2013, and 2014. Different N rates were applied to generate contrasting conditions of N availability in six *Japonica* cultivars (9915, 27123, Wuxiangjing 14, Wuyunjing 19, Yongyou 8, and Wuyunjing 24) and two *Indica* cultivars (Liangyoupei 9, YLiangyou 1). The SPAD values of the four uppermost leaves and LAI were measured from tillering to flowering growth stages. Two N indicators, leaf N accumulation (LNA) and plant N accumulation (PNA) were measured. The LAI estimated by LAI-2000 and LI-3050C were compared and calibrated with a conversion equation. A linear regression analysis showed significant relationships between Chl value and N indicators, the equations were as follows: PNA = (0.092 × Chl) − 1.179 (*R*^2^ = 0.94, *P* < 0.001, relative root mean square error (RRMSE) = 0.196), LNA = (0.052 × Chl) − 0.269 (*R*^2^ = 0.93, *P* < 0.001, RRMSE = 0.185). Standardized method was used to quantity the correlation between Chl value and grain yield, normalized yield = (0.601 × normalized Chl) + 0.400 (*R*^2^ = 0.81, *P* < 0.001, RRMSE = 0.078). Independent experimental data also validated the use of Chl value to accurately estimate rice N status and predict grain yield.

## Introduction

The crop canopy plays an important role in estimating the nitrogen (N) status of crops, which also could be used to predict grain yield (Hansen and Schjoerring, 2003; Fitzgerald et al., 2010; Lin et al., 2010). Several indicators, such as the leaf area index (LAI), canopy chlorophyll density (Chl), and surface radiation, can be used to elucidate the functions of the plant canopy (Chen and Cihlar, 1995). The LAI may be the most commonly used specific canopy indicator in crop research. It is a canopy structure–related factor, which is related directly to photosynthesis and biomass accumulation. This index has been used in numerous ecological models. For example, Knyazikhin et al. (2012) used LAI and fractions of photo-synthetically active radiation absorbed by vegetation from atmospherically active multi-angle imaging spectroradiometer data (Knyazikhin et al., 2012). Wu et al. (2014) reported that integrated LAI and leaf N accumulation (LNA) data performed better than did each parameter alone for crop model parameter optimization. The LAI was also used to optimize N dressing rates, as large volumes of LAI data are generated relatively easy (Wood et al., 2003).

Conventional methods of LAI measurement include sample weighing and leaf width to length ratio, both of which are time consuming, which makes the acquisition of large amounts of LAI data over space and time difficult (Wu et al., 2014). Optical instruments are widely used to measure the radiation transmitted through the canopy, by which the LAI can then be determined. Among various commercial optical instruments currently available for indirect in situ LAI measurement, the LAI-2000 plant canopy analyzer (LI-COR, Inc., Lincoln, NE, 2004) is the most widely used (Rico et al., 2009). Forest research showed that the LAI determined with the LAI-2000 is considerably smaller than the actual LAI measured directly (White et al., 2000). Stroppiana et al. (2006) also reported LAI-2000 tends to underestimate LAI in paddy rice when LAI > 1. These studies pointed out the need for proper validation when using the LAI-2000 to measure the LAI of plants.

Chlorophyll is another crucial factor in the estimation of plant N status (Hawkins et al., 2009). The SPAD meter (Minolta Camera Co., Osaka, Japan) is the most commonly used instrument for the determination of plant chorophyll and nitrogen concentration (Peng et al., 1993; Azia and Stewart, 2001). During the past two decades, rice N status monitored with the SPAD meter has been applied widely to determine N demand at different growth stages, with the goal of optimizing grain yield and N use efficiency (Peng et al., 1996). A real-time nondestructive modus was developed to determine leaf chlorophyll concentration from leaf color images captured with a portable digital camera, and to relate the results of leaf image analysis to SPAD-502 meter readings (Tian et al., 2011). Data from SPAD meters have been found to be correlated significantly with chlorophyll concentration, according to absorbance/transmittance measurements (Li et al., 2009). Moreover, the SPAD readings and normalized SPAD index has been proved to positively related to relative grain yield in rice (Yuan et al., 2016).

The responses of the same leaf, at different growth stages, to the N supply deserve attention when the SPAD meter is used to diagnose plant N status (Peng et al., 1996). Peng et al. (1993) proved that adjusting SPAD values for specific leaf weight (SLW; SPAD/SLW) improved the prediction of dry-weight N status. Unfortunately, the resulting single data point remained a relatively poor representation of rice status. In response, the Chl (Chl = SPAD_upper_ × LAI_green_) was proposed and used in estimating canopy chlorophyll status (Broge and Leblanc, 2001; Gitelson et al., 2005). Ciganda et al. (2009) also demonstrated the relationship between chlorophyll content in each leaf and total canopy Chl concentration, which were established based on the red-edge chlorophyll index: Chl_red−edge_ = (RNIR/R_red−edge_) (Ciganda et al., 2009).

The mid-season N status contributes to optimize crop N requirement. Normally, it can be determined by laboratory analysis of plant tissue nitrogen concentration (TNC), from chlorophyll concentration using chlorophyll meters, or from biomass measurements taken with plant gauge (Stevens et al., 2008). These methods require intensive sampling to obtain representative values. Tissue N accumulation (TNA), as an indicator of tissue N content and tissue weight, reflects not only tissue N status, but also vegetation coverage at various crop growth stages (Zhou et al., 2006).

In general, TNA is regarded as a better indicator of grain yield in rice than TNC or biomass alone (Ntamatungiro et al., 1999), which is significant for crop N diagnosis, real-time fertilization, and production prediction. TNA refers to the amount of total N in the plant canopy per unit area, and can be calculated as the product of dry biomass and TNC. TNA is also known to be a better indicator of N stress in rice than TNC, and can be considered as a potentially better indicator of mid-season N status and grain yield prediction (Nguyen et al., 2006; Ata-Ul-Karim et al., 2016).

The objectives of this research were: to analyze the relationship between LAI-2000 and LI-3050C based LAI, to develop Chl based models for estimating PNA and LNA, and to construct a proper model based on newly normalized Chl for predicting grain yield in irrigated lowland rice.

## Materials and methods

### Field experiments

Six field experiments were conducted from 2007 to 2009 at Nanjing Agricultural Experimental Station, from 2013 to 2014 at Wujiang, Zhangjiagang and Rugao Experimental Stations, Jiangsu Province, China. These regions receive more than 2,000 h of sunshine and 1000 mm rainfall annually, with an average temperature of 15.7°C. Rice/wheat (*Triticum aestivum* L.) rotation is the typical cropping system in these stations.

A randomized complete block design was used to arrange the combinations of rice cultivars and N treatments in all of the experiments, at a plant density of 5.33 × 10^5^ plants ha^−1^. Plot areas were 27 m^2^ (4.5 × 6 m) in experiments 1–3, 30 m^2^ (5 × 6 m) in experiment 4, and 42 m^2^ (6 × 7 m) in experiments 5-6. During plowing, phosphorus and potassium fertilizers were applied at the rate of 135 kg ha^−1^ P_2_O_5_ [Ca(H_2_PO_4_)_2_] and 190 kg ha^−1^ K_2_O (KCl), respectively. The N fertilizer used urea with an N content of 46%. The distribution of total N at different growth stages was 50% before transplanting, 20% at tillering, and 30% at booting (de Siriwardene et al., 1966). Irrigation was applied at 6-day intervals, except during rainfall events. Detailed information is shown in Table 1.

**Table 1:** Basic information about the six field experiments used in the study.

**Experiment No**.	**Location**	**Cultivar**	**N rate (Kg ha^−1^)**	**Sampling stage (date)**	**Soil classification**
Experiment 1 2007	Nanjing (31°56′N, 118°59′E)	9915, 27123 *(Japonica)*	N0(0) N1(120) N2(240) N3(360)	TI(17-July) SE(29-July) PI (12-August) BT(18-August) HD(25-August)	Yellow white soil
Experiment 2 2008	Nanjing (31°56′N, 118°59′E)	27123, WXJ-14 *(Japonica)*	N0(0) N1(130) N2(260) N3(390)	TI(20-July) SE(01-August) PI (16-August) BT(22-August) HD(30-August)	Yellow white soil
Experiment 3 2009	Nanjing (31°56′N, 118°59′E)	WXJ-14 *(Japonica)*, LYP-9 *(Indica)*	N0(0) N1(120) N2(240) N3(360)	TI (19–July) SE (04–August) BT(22–August) HD (25–August)	Yellow white soil
Experiment 4 2013	Wujiang (31°15′N, 120°72′E)	WYJ-19, YY-8 *(Japonica)*	N0(0) N1(90) N2(180) N3(240) N4(360)	TI(18-July) SE(31-July) PI (13-August) BT(20-August) HD(27-August) FL(4-September)	Clay loam soil
Experiment 5 2014	Zhangjiagang (31°87′N,120°77′E)	WYJ-19, YY-8 *(Japonica)*	N0(0) N1(90) N2(180) N3(240) N4(360)	TI(23-July) SE(01-August) HD(29-August)	Loam soil
Experiment 6 2014	Rugao (32°27′N,120°76′E)	WYJ-24 *(Japonica)*, YLY-1*(Indica)*	N0(0) N1(150) N2(225) N3(300) N4(375)	TI(18-July) SE(30-July) PI(06-August) BT(16-August) HD(26-August)	Loam soil

*Rice cultivars: Wuxiangjing-14 (WXJ-14), Liangyoupei-9 (LYP-9), Wuyunjing-19 (WYJ-19), Yongyou-8 (YY-8), Wuyunjing-24 (WYJ-24), YLiangyou-1 (YLY-1)*.

*Rice growth stages: tillering (TI), stem elongation (SE), panicle initiation (PI), booting (BT), heading (HD), flowering (FL)*.

Experiments 1, 2, and 4 were conducted to establish models for estimating N status and predicting grain yield. Experiment 6 was conducted to compare LAIs measured directly by LI-3050C and indirectly by LAI-2000. LAI calibration was conducted using the independent experimental data of experiment 3. The predicting accuracy of models for estimating N status and predicting grain yield was evaluated from experiments 5 and 6.

### Sampling and measurements

In each experiment, five hills from each plot were sampled for growth analysis at different growth stages (Table 1). Rice plants were uprooted manually and cut at the ground level to measure N concentration. Fresh plants were separated into green leaf blade (leaf) and culm plus sheath (stem) portions, heated for 30 min at 105°C to halt metabolism, and dried at 80°C in a forced-draft oven until they reached a constant weight. Plant dry matter was determined, and the samples were pulverized before passage through a 1-mm sieve in a Wiley mill. Then, the samples were stored in plastic bags at room temperature until further chemical analysis. Samples (0.2 g) were dried and ground, and digested using a mixture of H_2_O_2_ and H_2_SO_4_; the N content was then determined using a continuous-flow auto-analyzer (BRAN + LUEBBE AA3, Germany). Grain yield was determined for each plot at maturity by harvesting plants in a 2 m^2^ area in each plot, at a moisture content of 13.5%.

Before sampling, 10 plants were selected randomly in each plot and SPAD values were taken from four upper fully expanded leaves (Peng et al., 1993) with a SPAD-502 meter. And the SPAD values were recorded by inserting the leaf portion up to 1/3, 1/2, and 2/3 into the slit of the SPAD meter. Finally, the average SPAD value of the 10 plants was used to represent the SPAD reading in the plot.

The LAI determined with the LI-3050C leaf area meter (LI-COR, Lincoln, U.S.) served as the actual LAI of the corresponding plot during the procedure of handling samples. Before sampling, LAI-2000 (LI-COR, Lincoln, U.S.) was used to non-destructively estimate LAI by measuring at dusk or dawn and consisted of a sequence of readings (one above and four below the rice canopy) taken three times at each plot with a 90° view cap (Sone et al., 2009).

### Data analysis

#### Plant and leaf N accumulation

Plant N accumulation (PNA) at each growth stage (Samborski et al., 2009) was calculated as follows:

(1) $$\begin{array}{r} \text{PNA}=\text{LDM }\times \text{ LNC}+\text{SDM }\times \text{ SNC} \end{array}$$

where LDM and SDM (kg ha^−1^) are the dry matters weight of leaves and stem, while LNC and SNC (N %) are the N concentrations of the leaves and stem, respectively.

Leaf N accumulation (LNA) was calculated with LDM and LNC as follows:

(2) $$\begin{array}{r} \text{LNA}=\text{LDM }\times \text{ LNC} \end{array}$$

#### Canopy chlorophyll density

Canopy chlorophyll density (Chl) at each growth stage was calculated as follows:

(3) $$\begin{array}{r} \text{Chl}=\text{LA}{\text{I}}_{\text{green}}\;\times \text{ SPA}{\text{D}}_{\text{upper}} \end{array}$$

where SPAD_upper_ is the chlorophyll concentration of the upper leaves, and LAI_green_ represents the green LAI determined by LI-3050C or manually (Gitelson et al., 2003).

#### Normalized yield and Chl

To predict grain yield, we used normalized method to calculate normalized Chl and yield at each growth stage:

(4) $$\begin{array}{r} \text{NormalizedChl}=\text{Chl}/\text{Ch}{\text{l}}_{\text{max}} \end{array}$$

(5) $$\begin{array}{r} \text{NormalizedYield}=\text{Yield}/\text{Yiel}{\text{d}}_{\text{max}} \end{array}$$

where Chl_max_ is the maximum Chl of each growth stage, Yield_max_ is the maximum of grain yield among all yields in different cultivars (Saad et al., 2004).

#### Model evaluation

Statistical analyses were performed on the data pooled over two seasons using Statistical Product and Service Solutions software (SPSS 20.0; IBM, U.S.). Exponential regression equations were fitted with PNA, LNA, grain yield and Chl, recorded with different instruments at different growth stages. In order to validate these prediction models, the relative root mean square error (RRMSE, %) (Wallach and Goffinet, 1989) was used to test the compliance of model predicted value with measured value.

## Results

### Comparison between LAI-2000 and LI-3050C

The operating principles of the LAI-2000 and LI-3050C meters differ; we found significant differences between LAI-2000 values (0.2–4.8) and LI-3050C values (0.5–8.0). The LAI-2000 values were generally lower than those from LI-3050C. Overall, there was a close statistical relationship between these LAI values measured with two instruments. Higher coefficients of determination (*R*^2^) were observed primarily in vegetative growth stages: stem elongation, panicle initiation, and booting. Moreover, lower positive correlation between LAI-2000 and LI-3050C was found at the tillering stage (Table 2) (*R*^2^ = 0.501, *P* < 0.001). Difference between the two instruments might be attributed to less-expanded leaves and light reflection of water.

**Table 2:** Correlations of LAI values between LI-3050C and LAI-2000 at different growth stages in experiment 6.

**Growth stage**	**Data (*n*)**	**Regression equation**	***R^2^***
Tillering (TI)	30	LI = 1.02 × LAI + 0.52	0.50^***^
Stem elongation (SE)	30	LI = 1.25 × LAI – 0.06	0.86^***^
Panicle initiation (PI)	30	LI = 1.53 × LAI – 0.30	0.82^***^
Booting (BT)	30	LI = 1.84 × LAI – 0.65	0.85^***^
Heading (HD)	30	LI = 1.76 × LAI – 0.28	0.78^***^
Entire growth stages	150	LI = 1.78 × LAI – 0.81	0.87^***^

“LI” is the actual LAI value determined by LI-3050C; “LAI” is the estimated LAI value with LAI-2000.

*** *F-test statistical significance at 0.001 probability level*.

Little variation was observed between LAI-2000 and LI-3050C values during the late growth period, except that due to severe weather conditions. Thus, to improve the accuracy of LAI monitoring, a linear model was adopted to reconstruct the relationship between LAI-2000 and LI-3050C values over growth stages. The linear model (LI = 1.78 × LAI – 0.81; *R*^2^ = 0.87, *P* < 0.001, Table 2) confirmed that LAI-2000 values were smaller than those from the LI-3050C meter. The model can calibrate the LAI values measured with LAI-2000 and improve the accuracy and efficiency.

### Trends of Chl (SPAD_upper_ × LAI_green_), PNA and LNA

**Figure 1:**
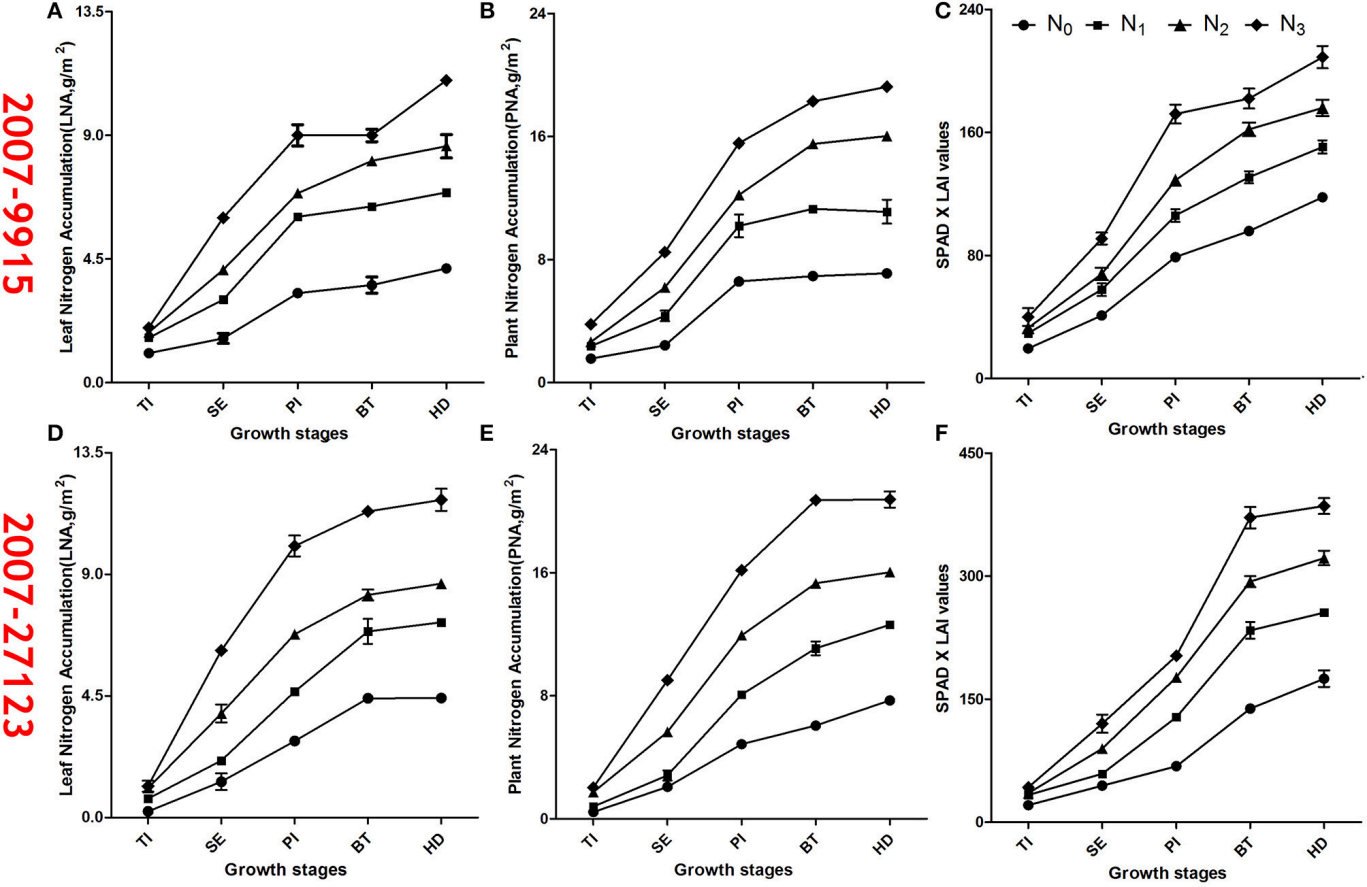
Time series changes of SPAD × LAI values, LNA and PNA (±SE) for two rice cultivars: 9,915 used in experiment 1 with N treatments of 0, 120, 240, and 360 kg ha^−1^; and 27,123 used in experiment 2 with N treatments of 0, 130, 260, and 390 kg ha^−1^. The LAI was estimated with LAI-2000.The sub title is the year of experiment + cultivar. 2007–9,915: **(A)** is the number of “LNA” figure, **(B)** is the number of “PNA” figure, **(C)** is the number of “Chl” figure; 2007–27,123: **(D)** is the number of “LNA” figure, **(E)** is the number of “PNA” figure, **(F)** is the number of “Chl” figure.

The Chl data were averaged over three replications of data measured destructively and non-destructively, during the period from TI to HD (Figure 1). Consistent differences in Chl were observed among N treatments, in the order N3 > N2 > N1 > N0. For all N treatments, however, increases were observed from TI to SE; sharp increases were seen from SE to PI; but consistent, increases were observed from PI to BT; and values remained relatively constant from BT to heading (HD). The values increased following PI. However, the rates of increase of all indices were lower than those at earlier growth stages. The trends of PNA and LNA were similar to those for Chl. Chl values ranged from 20 to 410 (Figures 1C–F), PNA values ranged from 0.5 to 22 g m^−2^ (Figures 1B,E), and LNA values ranged from 0.4 to 12 g m^−2^ (Figures 1A,D). The turning point over all growth stages was between PI (slope = 1.53) and BT (slope = 1.84). The slopes were lower before TI (slope = 1.02, SE: slope = 1.25), and after HD (slope = 1.76).

To explore the relationships of Chl with PNA and LNA, we analyzed the changes of these indices at the various growth stages. Even at a relatively early stage, the Chl and N indicators could be very different. Differences among N treatments at the panicle initiation stage were obvious (Table 3); thus, the use of absolute SPAD, LAI, and Chl values to estimate crop N status was appropriate. At panicle initiation stage, the differences were highly significant.

**Table 3:** Statistical analysis of Chl values, LNA and PNA at the panicle initiation stage under different N treatments in experiments 1 and 2.

**Experiment No**.	**Cultivar**	**N treatment**	**Chl**	**PNA (g m^−2^)**	**LNA (g m^−2^)**
Experiment 1 2007	9915	N0	95.88^d^	6.93^d^	3.55^d^
		N1	130.85^c^	11.30^c^	6.41^c^
		N2	162.03^b^	15.52^b^	8.07^b^
		N3	182.45^a^	18.29^a^	9.41^a^
	27123	N0	101.64^d^	6.80^d^	3.93^d^
		N1	125.12^c^	10.37^c^	6.04^c^
		N2	151.57^b^	14.10^b^	7.50^b^
		N3	169.54^a^	17.34^a^	8.65^a^
Experiment 2 2008	WXJ-14	N0	68.45^d^	4.86^d^	2.83^d^
		N1	128.25^c^	8.09^c^	4.66^c^
		N2	176.76^b^	11.94^b^	6.78^b^
		N3	203.04^a^	16.17^a^	10.05^a^
	27123	N0	58.06^c^	4.43^d^	2.53^c^
		N1	115.98^bc^	7.52^c^	4.36^c^
		N2	181.01^ab^	11.29^b^	6.61^b^
		N3	218.61^a^	15.37^a^	9.45^a^

a, b, c, d *mean F-test statistical significance at the 0.001 probability level*.

### Correlations of Chl (SPAD_upper_ × LAI_green_) with PNA and LNA

Chl could be used to diagnose the N status of rice, and N accumulation is a commonly used indicator of N status. To compare the relationships of Chl to PNA and LNA, we performed linear analyses at individual growth stages and overall growth stages, and the most stable and generally applicable duration model was developed (Table 4). Linear correlation analysis indicated that PNA and LNA in rice were related positively to Chl, with 0.001 significance levels at different growth stages. Compared with the relationships of SPAD and LAI values to N accumulation, we found that the relationships of Chl to PNA and LNA were linear at all stages, with higher *R*^2^ values (*R*^2^ > 0.82, *P* < 0.001). The SPAD and LAI values had multi-linear relationships with PNA and LNA, particularly with LNA.

**Table 4:** Correlations of SPAD, LAI, Chl with PNA and LNA at different growth stages in experiments 1, 2, and 4.

**Experiment No**.	**Growth stage**	**Nitrogen accumulation**	**SPAD**		**LAI**		**Chl**	
			**Regression model**	**R2**	**Regression model**	**R2**	**Regression model**	**R2**
Experiment 1 2007	TI	PNA	E	0.68^*^	L	0.69^*^	L	0.90^***^
		LNA	P	0.69	P	0.71^*^	L	0.90^***^
	SE	PNA	L	0.73^**^	Q	0.77^**^	L	0.92^***^
		LNA	Q	0.74^**^	L	0.76^**^	L	0.91^***^
	PI	PNA	Q	0.74^**^	E	0.79^**^	L	0.97^***^
		LNA	L	0.68^*^	P	0.76^**^	L	0.97^***^
	BT	PNA	P	0.71^**^	Q	0.76^**^	L	0.99^***^
		LNA	L	0.73^*^	Q	0.73^**^	L	0.99^***^
	HD	PNA	Q	0.72^**^	L	0.71^*^	L	0.95^***^
		LNA	Q	0.75^*^	L	0.76^**^	L	0.96^***^
	Entire stages	PNA	–	–	–	–	L	0.94^***^
		LNA	–	–	–	–	L	0.92^***^
Experiment 2 2008	TI	PNA	L	0.61^*^	P	–	L	0.86^***^
		LNA	L	0.59^*^	L	–	L	0.83^***^
	SE	PNA	E	0.69^**^	L	0.76^**^	L	0.82^***^
		LNA	E	0.67^*^	P	0.84^**^	L	0.85^***^
	PI	PNA	L	0.70^**^	L	0.76^**^	L	0.95^**^
		LNA	P	0.71^**^	L	0.81^**^	L	0.92^***^
	BT	PNA	Q	0.80^**^	E	0.79^**^	L	0.95^***^
		LNA	L	0.73^**^	Q	0.81^**^	L	0.97^***^
	HD	PNA	E	0.79^**^	Q	0.64^*^	L	0.98^***^
		LNA	P	0.77^**^	L	0.79^**^	L	0.94^***^
	Entire stages	PNA	–	–	–	–	L	0.97^***^
		LNA	–	–	–	–	L	0.98^***^
Experiment 4 2013	TI	PNA	Q	0.84^**^	L	0.56^*^	L	0.89^**^
		LNA	Q	0.84^**^	L	0.52^*^	L	0.90^**^
	SE	PNA	L	0.51^*^	E	0.73^*^	L	0.94^***^
		LNA	L	0.53^*^	E	0.79^**^	L	0.93^***^
	PI	PNA	P	0.72^**^	L	0.74^*^	L	0.97^***^
		LNA	L	0.68^**^	P	0.86^**^	L	0.94^***^
	BT	PNA	L	0.72^*^	Q	0.78	L	0.92^***^
		LNA	E	0.74^**^	L	0.67^*^	L	0.91^***^
	HD	PNA	Q	0.86^**^	E	0.83^**^	L	0.95^***^
		LNA	Q	0.79^**^	P	0.71^*^	L	0.96^***^
	FL	PNA	L	0.68^*^	L	0.13^ns^	L	0.97^***^
		LNA	E	0.63^*^	E	0.2^ns^	L	0.98^***^
	Entire stages	PNA	–	–	–	–	L	0.92^***^
		LNA	–	–	–	–	L	0.88^***^

Regression models: linear (L), quadratic (Q), exponential (E), power (P);

ns and “–” means non-significance;

* F-test statistical significance at 0.05 probability level;

** F-test statistical significance at 0.01 probability level;

*** *F-test statistical significance at 0.001 probability level*.

Table 4 shows regression terms and parameters for corresponding curves. *R*^2^ values (*R*^2^ > 0.9) for the most of relationships between LNA and Chl were higher than those for the relationship between PNA and Chl (*R*^2^ > 0.84), like the TI stage in 2008. In addition, intercepts and slopes tended to vary with growth stages; thus, different regression coefficients were needed to represent N accumulation at given stages. *R*^2^ values were highest at PI (*R*^2^ > 0.92, *P* < 0.001), indicating that this stage was the best time to apply top-dressing fertilizer.

**Figure 2:**
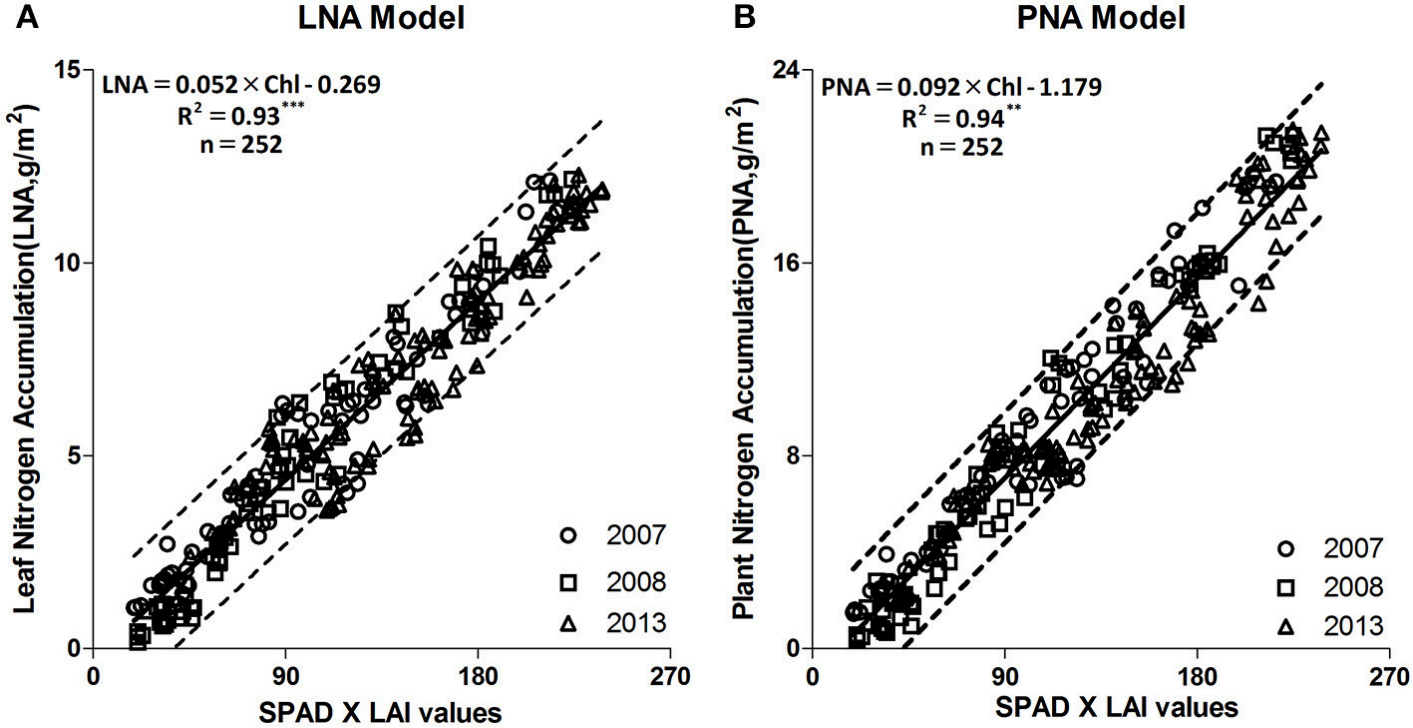
Linear regression fitted between Chl and PNA, LNA at different years (2007, 2008, and 2013). **(A)** is the number of the “LNA Model” figure, **(B)** is the number of the “PNA Model” figure. ^**^F-test statistical significance at 0.01 probability level; ^***^F-test statistical significance at 0.001 probability level.

To create a new model for monitoring N status, the relationships of Chl to PNA and LNA were analyzed based on data from experiments 1, 2, and 4 for five rice cultivars. The *R*^2^ values for these relationships were 0.93 and 0.94, respectively (*P* < 0.001; Figure 2). The slopes for the regression equations representing Chl and LNA in 2007, 2008, and 2013 were 0.051, 0.057, and 0.053, respectively. The equations to estimate PNA and LNA were: PNA = (0.092 × Chl) – 1.179 (*R*^2^ = 0.94^***^, *n* = 252, Figure 2B); LNA = (0.052 × Chl) – 0.269 (*R*^2^ = 0.93^***^, *n* = 252, Figure 2A).

### Correlation between Chl (SPAD_upper_ × LAI_green_) and yield

Canopy interception, evapotranspiration, photosynthesis, and grain yield are directly proportional to LAI and chlorophyll. A comprehensive analysis of the correlations of SPAD, LAI, and Chl with grain yield over years, growth stages, and cultivars are presented in Table 5. This analysis revealed that regression of grain yield on Chl produced high *R*^2^ values (*R*^2^ > 0.88, *P* < 0.001, RRMSE < 0.19); regression of grain yield on SPAD and LAI values also produced high values (SPAD: 0.89 > *R*^2^ > 0.60, *P* < 0.001, RRMSE > 0.21; LAI: 0.97 > *R*^2^ > 0.66, *P* < 0.001, RRMSE > 0.23). The lowest *R*^2^ values for yield, Chl, SPAD, and LAI values were at BT, except in 2008, when values were lowest at PI (Chl: 0.78 < *R*^2^ < 0.9, *P* < 0.001, 0.14 < RRMSE < 0.19; SPAD: *R*^2^ > 0.88, *P* < 0.001, RRMSE < 0.19; LAI: 0.96 > *R*^2^ > 0.64, *P* < 0.001, 0.43 > RRMSE > 0.23). Compared with the SPAD and LAI values, we concluded that Chl improved yield prediction across diverse environmental conditions. The *R*^2^-values for the relationships of yield to SPAD and LAI values were inconsistent, reflecting instability, suggesting that the use of a single index to predict rice yield is risky.

**Table 5:** Correlations of SPAD, LAI and Chl with grain yield at different growth stages in experiments 1, 2, and 4.

**Experiment No**.	**Cultivar**	**Growth stage**	**SPAD**		**LAI**		**Chl**	
			**R2**	**RRMSE**	**R2**	**RRMSE**	**R2**	**RRMSE**
Experiment 1 2007	9,915	TI	0.77^**^	0.5	0.85^**^	0.36	0.93^***^	0.17
		SE	0.81^**^	0.7	0.83^**^	0.68	0.92^***^	0.14
		PI	0.73^*^	0.41	0.89^**^	0.51	0.94^***^	0.19
		BT	0.78^**^	0.51	0.66^*^	0.4	0.90^***^	0.29
		HD	0.81^**^	0.32	0.88^**^	0.95	0.92^***^	0.18
	27,123	TI	0.81^**^	0.36	0.92^***^	0.5	0.94^***^	0.19
		SE	0.77^*^	0.51	0.83^**^	0.7	0.97^***^	0.16
		PI	0.81^**^	0.4	0.86^**^	0.41	0.99^***^	0.11
		BT	0.67^*^	0.43	0.64^*^	0.51	0.78^**^	0.51
		HD	0.83^**^	0.59	0.92^***^	0.32	0.91^***^	0.22
	Normalized		0.79^**^	0.49	0.83^**^	0.53	0.88^***^	0.21
Experiment 2 2008	WXJ-14	TI	0.79^**^	0.4	–	0.47	0.92^***^	0.18
		SE	0.77^**^	0.31	0.84^**^	0.46	0.96^***^	0.15
		PI	0.75^*^	0.4	0.99^***^	0.43	0.87^***^	0.28
		BT	0.75^*^	0.31	0.85^**^	0.59	0.96^***^	0.16
		HD	0.83^**^	0.21	0.76^*^	0.49	0.98^***^	0.13
	27,123	TI	0.77^*^	0.14	0.09^ns^	–	0.98^***^	0.12
		SE	0.79^**^	0.29	0.73^**^	0.31	0.97^***^	0.14
		PI	0.8^**^	0.56	0.88^**^	0.21	0.90^***^	0.2
		BT	0.84^**^	0.52	0.96^***^	0.14	0.99^***^	0.1
	Normalized		0.81^**^	0.41	0.84^**^	0.29	0.86^***^	0.24
Experiment 4 2013	WYJ-19	TI	0.79^**^	0.5	0.81^**^	0.56	0.94^***^	0.15
		SE	0.49^ns^	–	0.87^***^	0.52	0.94^***^	0.16
		PI	0.83^**^	0.28	0.97^***^	0.43	0.97^***^	0.13
		BT	0.72^*^	0.57	0.84^**^	0.52	0.88^***^	0.26
		HD	0.81^**^	0.46	0.96^***^	0.41	0.93^***^	0.19
		FL	0.82^**^	0.31	0.76^*^	0.5	0.97^***^	0.14
	YY-8	TI	0.81^**^	0.21	0.79^**^	0.92	0.87^***^	0.22
		SE	0.33^ns^	0.14	0.98^***^	0.18	0.95^***^	0.17
		PI	0.6^*^	0.29	0.86^***^	0.33	0.92^***^	0.19
		BT	0.71^*^	0.56	0.95^***^	0.28	0.90^***^	0.24
		HD	0.79^**^	0.18	0.76^*^	0.57	0.91^***^	0.13
		FL	0.89^***^	0.33	0.86^***^	0.46	0.88^***^	0.37
	Normalized		0.75^*^	0.28	0.81^**^	0.4	0.92^***^	0.24

*ns and “–” mean non-significance*.

* F-test statistical significance at 0.05 probability level;

** F-test statistical significance at 0.01 probability level;

*** *F-test statistical significance at 0.001 probability level*.

**Figure 3:**
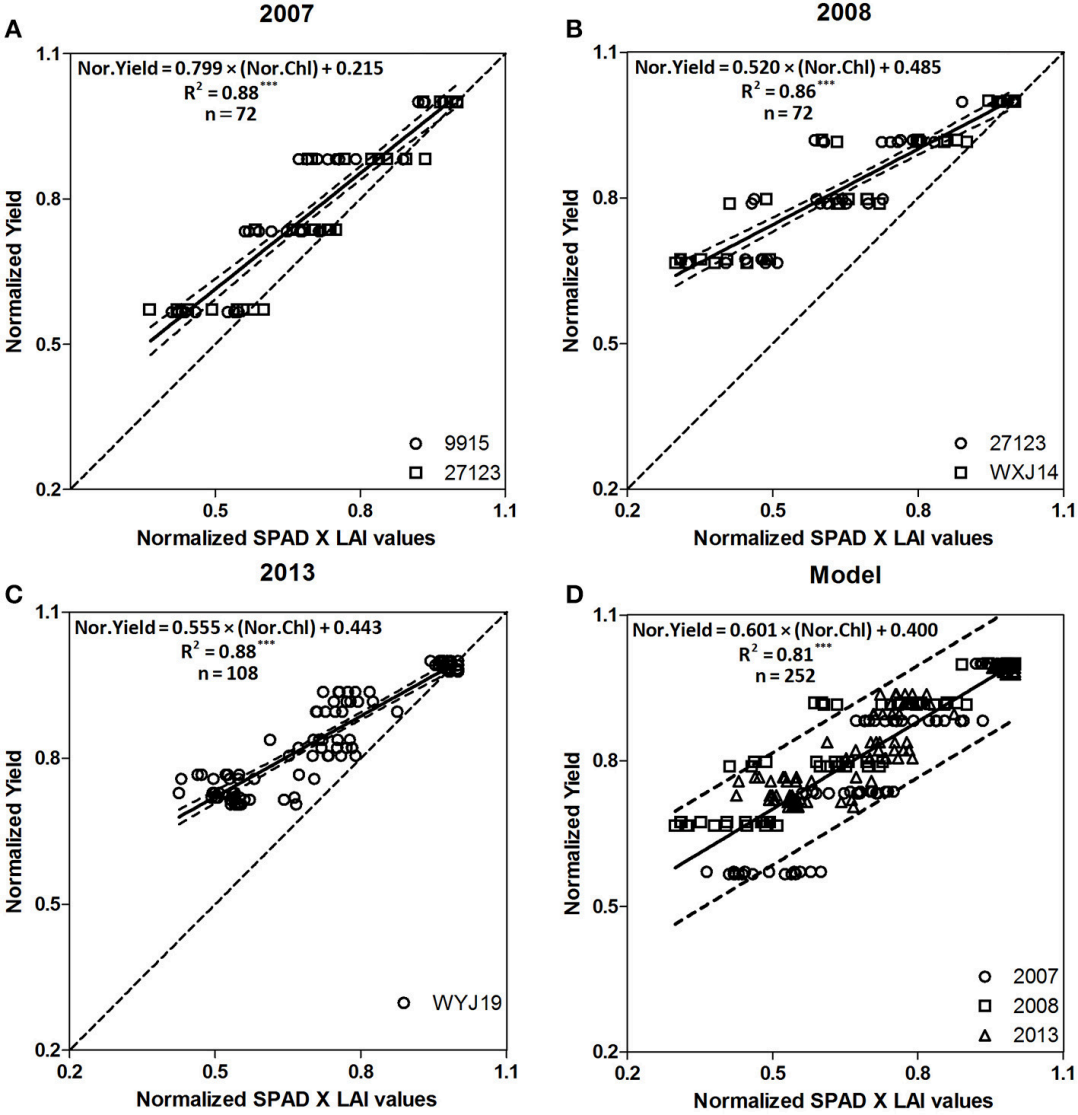
Relationships between normalized Chl (Normalized SPAD × LAI values) and normalized yield of five Japonica cultivars (2007: 9,915, 27,123; 2008: 27,123, WXJ-14; 2013: WYJ-19, YY-8) from tillering to flowering growth stages. The solid line denotes the linear regression and the dotted line indicates a line inclined at 45° to the axes. **(A)** is the number of the “2007” figure, **(B)** is the number of the “2008” figure, **(C)** is the number of the “2013” figure and **(D)** is the number of the “Model” figure. ^***^F-test statistical significance at 0.001 probability level.

To improve the accuracy of grain yield prediction, the new models were established using normalized method. Normalization was used to standardize variability in yield and Chl for each of 3 years (2007: normalized yield = [0.799 × normalized Chl] + 0.215, *R*^2^ = 0.88, *P* = 0.001, Figure 3A; 2008: normalized yield = [0.520 × normalized Chl] + 0.485, *R*^2^ = 0.86, *P* = 0.001, Figure 3B; 2013: normalized yield = [0.555 × normalized Chl] + 0.443, *R*^2^ = 0.88, *P* = 0.001, Figure 3C). In addition, a comprehensive model for grain yield prediction was developed based on normalized data from the three experiments (normalized yield = [0.601 × normalized Chl] + 0.400, *R*^2^ = 0.81, *P* = 0.001, Figure 3D).

## Discussion

### LAI feature model

**Figure 4:**
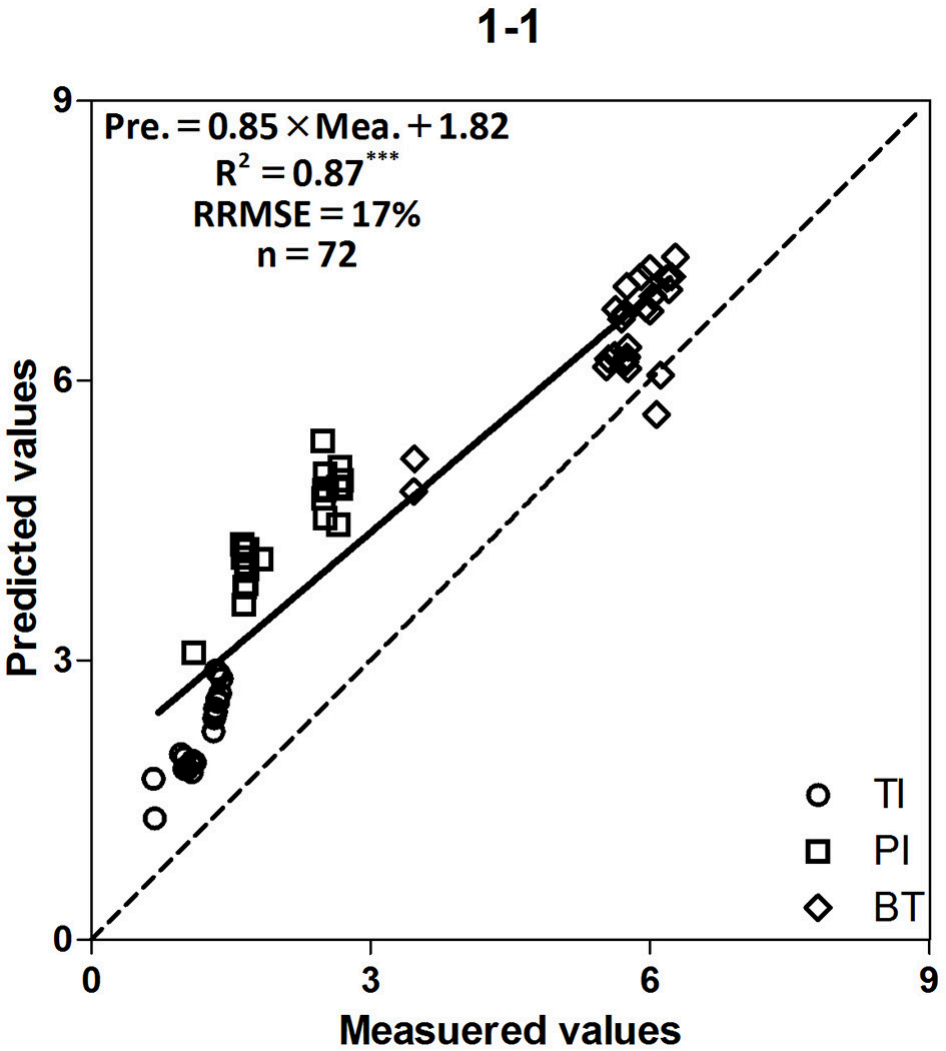
Relationship between measured and predicted LAI values of two Japonica cultivars at the tillering, panicle initiation and booting stages. The solid line denotes the linear regression and the dotted line is a line inclined at 45° to the axes. ^***^F-test statistical significance at 0.001 probability level.

This study was designed to evaluate the accuracy and range of reliability of LAI values measured by LAI-2000, compared with those from the LI-3050C meter. Previous studies concluded that the LAI-2000 tends to overestimate the LAI when values < 1, and underestimate the LAI when values > 1 (Gower et al., 1999; Stroppiana et al., 2006). In this study, the data points tended to aggregate upper the 1:1 line for LAI > 1 (Table 2). The results also provided a satisfactory fit between measured and predicted values with a linear equation (Figure 4). Although LAI-2000 values were lower than the actual LAI values, the LAI-2000 meter provides a rapid, real-time, and non-destructive approach to monitoring LAI (Rico et al., 2009). The LI-3050C meter generates actual values, but measurement is destructive and time-consuming. Therefore, for rapid, non-destructive estimation of LAI, we used the LI-3050C values and model to correct the LAI-2000 values.

### TNA feature models

**Figure 5:**
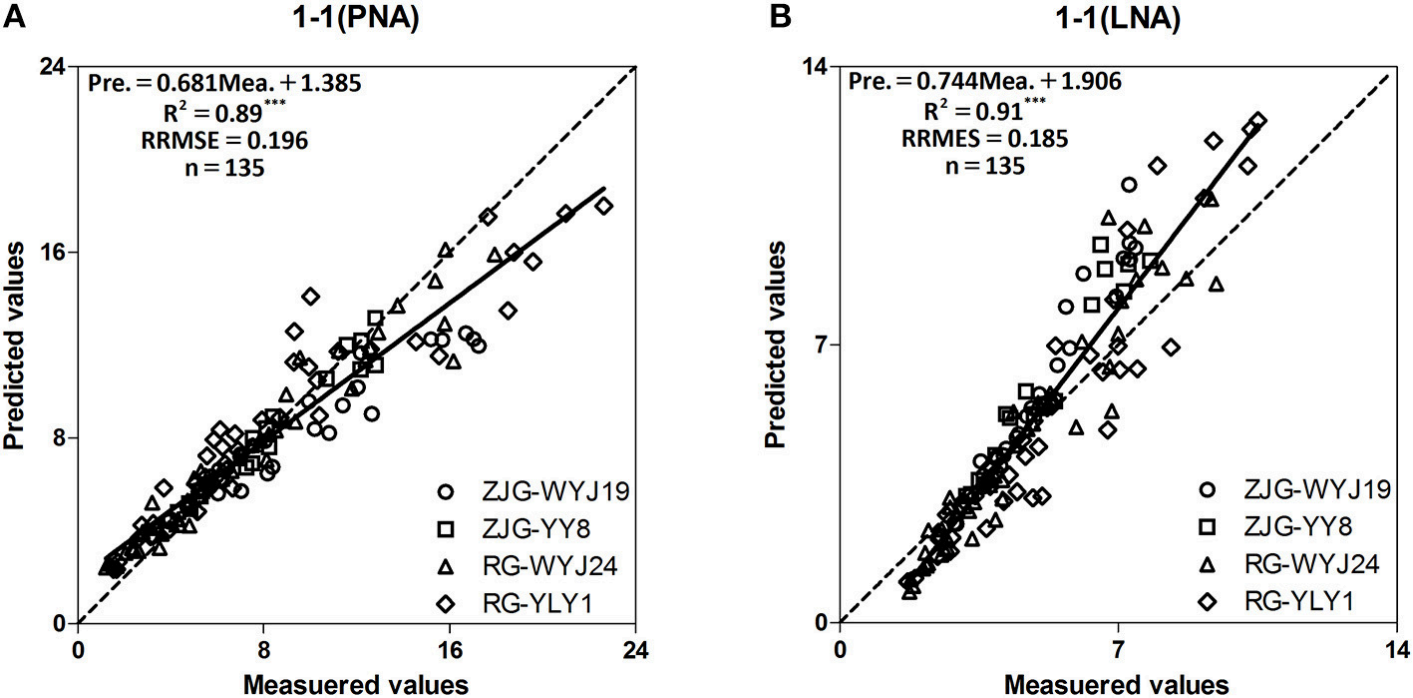
Relationships between measured and predicted PNA and LNA values of four rice cultivars (WYJ-19, YY-8, WYJ-24, Japonica; YLY-1, Indica; ZJG, Zhangjiagang; RG, Rugao) from stem elongation (SE) to booting (BT) growth stages. The solid line denotes the linear regression and the dotted line is a line inclined at 45° to the axes. **(A)** is the number of “1-1(PNA)” figure; **(B)** is the number of “1-1(LNA)” figure. ^***^F-test statistical significance at 0.001 probability level.

The results demonstrated that the relationships of Chl to PNA and LNA were stable across years, cultivars and growth stages, and that both PNA and LNA could be used to diagnose N deficiency in rice plants (Ntamatungiro et al., 1999). To test the accuracy of the model, we developed a 1:1 correlation diagram between measured and predicted values for N accumulation. The R^2^, RRMSEs of PNA and LNA were 0.89, 0.91; and 19.6% (Figure 5A), 18.5% (Figure 5B), respectively. The models that describe rice N status based on Chl have been shown to be highly accurate.

Previous studies focused on the canopy levels and showed that NDVI and other VIs were correlated strongly with N, and could distinguish different N treatments (Yan et al., 2006; Zhu et al., 2007; Liu et al., 2017). Although it would likely help to validate the concept of a need-based N application strategy that relies on TNA levels estimated from VIs, the expense of the instruments and complex analyses required make it difficult to promote the application of canopy spectral indices (Ntamatungiro et al., 1999). Peng et al. (2007) used a chlorophyll meter to monitor rice N status, and develop site-specific N dressing management method for resulting in higher grain yield with lower N dressing rates (Liu et al., 2009). However, single SPAD or LAI values cannot fully reflect the canopy dynamics and N status of crops (Wang et al., 2003; Zhong et al., 2006; Chen et al., 2014).

To solve this problem, we used the combination of SPAD and LAI values to predict TNA at the canopy levels. The Chl was found to be reliable and effective for the acquisition of PNA and LNA data, and that Chl values vary only slightly among rice cultivars. Therefore, the estimation of LNA and PNA should base on a theoretical framework and the practical application of real-time monitoring in rice. Results from this study demonstrated that Chl can provide comprehensive information about leaf and total PNA, accurately indicate the N status of rice. Comparison with existing VIs showed that the methods proposed in this paper are more convenient and inexpensive for PNA and LNA evaluation. For Chl, we considered chlorophyll content and the impacts of different growth stages while making full use of N data obtained from various experiments. Future work should use more independent data to validate and adjust the model, and develop the undetermined relationships of VIs to Chl.

### Yield feature model

**Figure 6:**
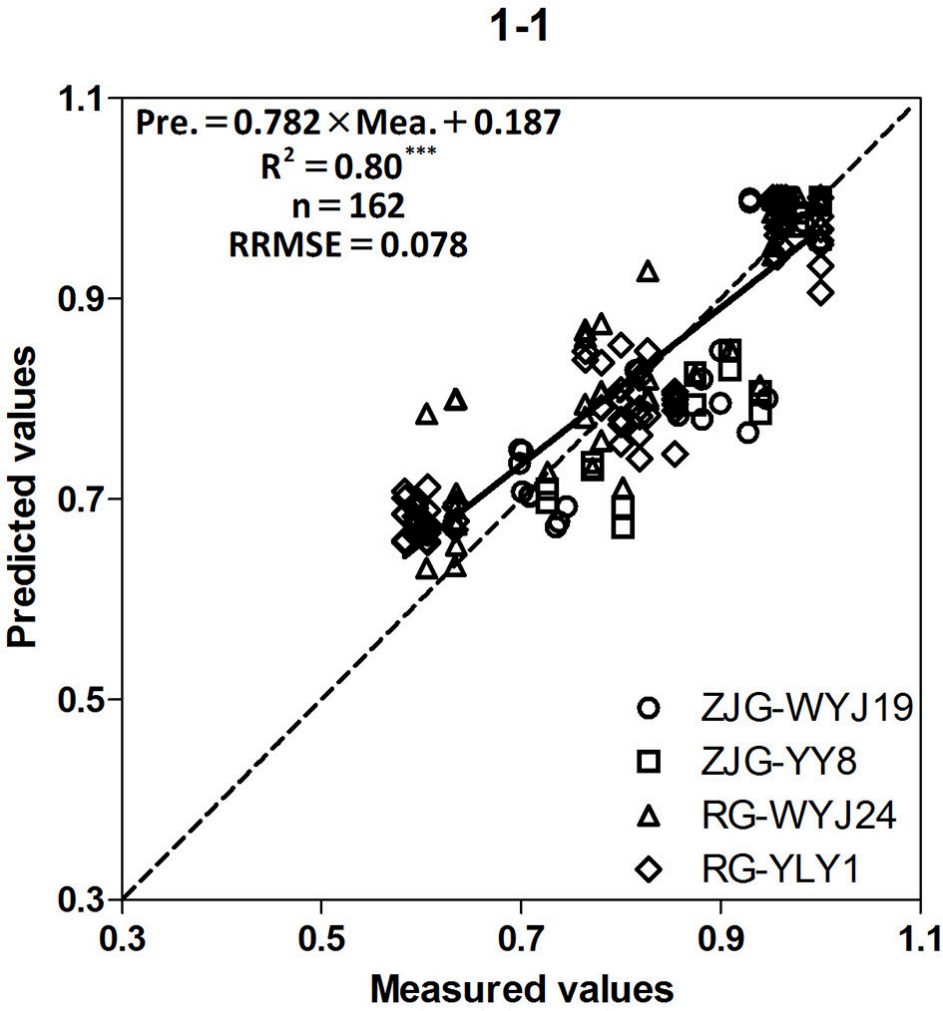
Relationship between measured and predicted normalized yield of four rice cultivars (WYJ-19, YY-8, WYJ-24, Japonica; YLY-1, Indica; ZJG, Zhangjiagang; RG, Rugao) from stem elongation (SE) to heading (HD) growth stages. The solid line denotes the linear regression and the dotted line is a line inclined at 45° to the axes. ^***^F-test statistical significance at 0.001 probability level.

The prediction model of grain yield was tested on an independent dataset obtained from experiments 5 and 6 (Figure 6). Good agreement between the measured and predicted yields was based on SPAD-502 and LAI-2000 data. High *R*^2^ and low RRMSE values between predicted and measured yields suggest that this model could be used to satisfactorily predict grain yield. This type of model also has great potential for predicting grain yield in direct-seeding rice (Hamar et al., 1996).

Agricultural scientists predict yield at different growth stages based on their knowledge and experience (Bushong et al., 2016). They also make biometric measurements of crops to improve the predicting accuracy under the meteorological conditions that exist during the growing season, as well as historical observations of agro-meteorological conditions and their influence on yield (Jamieson and Semenov, 2000). In recent years, the method of visually evaluating crop condition in the preliminary and pre-final assessments of crop yield has been influenced considerably by a subjective approach (Thakur et al., 2010).

This study integrated the advantages of LAI and SPAD values, and then established the relationship between Chl and grain yield at different growth stages, fertility levels, cultivars, and climate conditions. The correlations between Chl and yield parameters differed significantly among growth stages. The correlation coefficients were highest at PI, which was also reported by Chen et al. (2014). Meanwhile, Hu et al. (2014) proposed that RSPAD values was significantly related to NNI, but the slopes of the response lines varied with varieties, growth stages, and leaf positions (Hu et al., 2014). Deviation will appear when using the correlation between relative grain yield and NNI. Therefore, caution is needed in using these relationships to determine rice N status at critical growth stages. To exclude the influence of these parameters, normalized method was used to standardize Chl and grain yield. Comprehensive analysis of the data from different growth stages showed that normalized Chl was correlated with normalized yield. The resulting model for monitoring grain yield of rice is easy to use and cost effectively.

## Conclusion

The research focused on the utility of Chl index to reflect LNA, PNA, and grain yield under different N rates and rice cultivars across growth stages. The results indicated that the Chl most effectively represented the variation of N status in rice, and also a great indicator of grain yield. Different experimental data responded to Chl similarly and showed strong relationships between N indicators and Chl in different years, which is a novel idea for diagnosing N nutrition in the crop canopy. The normalized Chl value was also correlated strongly with grain yield. In order to enlarge the application of these models and LAI-2000, the LAI estimated by LAI-2000 and LI-3050C were compared and calibrated with a conversion equation. These findings proved that Chl values could be of value at sensitive growth stages in irrigated lowland rice, contributing to the estimation of N status and prediction of yield. Further studies under diverse site conditions and rice cropping systems are desired to guarantee the widespread use of this index.

## Author contributions

XL performed experiments with support by KZ, ZZ, and ZY; ZL, YT, YZ, and WC provided advice and edited the manuscript; KZ and QC planned experiments and XL wrote the manuscript. All authors read and approved the final manuscript.

### Conflict of interest statement

The authors declare that the research was conducted in the absence of any commercial or financial relationships that could be construed as a potential conflict of interest. The reviewer MO and handling Editor declared their shared affiliation.
